# Preventing and Managing Cardiometabolic Risk: The Logic for Intervention

**DOI:** 10.3390/ijerph6102568

**Published:** 2009-09-30

**Authors:** Mark A. Pereira, Thomas E. Kottke, Courtney Jordan, Patrick J. O’Connor, Nicolaas P. Pronk, Rita Carreón

**Affiliations:** 1 Division of Epidemiology and Community Health, School of Public Health, University of Minnesota, Minneapolis, MN 55454-1015, USA; E-Mails: pereira@epi.umn.edu (M.A.P.); carr0172@umn.edu (C.J.); 2 HealthPartners Research Foundation, Minneapolis, MN 55440-1524, USA; E-Mails: Patrick.J.OConnor@HealthPartners.Com (P.J.O’C.); Nico.P.Pronk@HealthPartners.Com (N.P.P.); 3 JourneyWell, Minneapolis, MN 55425, USA; 4 America’s Health Insurance Plans, Washington, DC 20004, USA; E-Mail: rcarreon@ahip.org

**Keywords:** cardiometabolic risk, diet, physical activity, treatment, prevention, strategy

## Abstract

Cardiometabolic risk (CMR), also known as metabolic syndrome or insulin resistance syndrome, comprises obesity (particularly central or abdominal obesity), high triglycerides, low HDL, elevated blood pressure, and elevated plasma glucose. Leading to death from diabetes, heart disease, and stroke, the root cause of CMR is inadequate physical activity, a Western diet identified primarily by low intake of fruits, vegetables, and whole grains, and high in saturated fat, as well as a number of yet-to-be-identified genetic factors. While the pathophysiological pathways related to CMR are complex, the universal need for adequate physical activity and a diet that emphasizes fruits and vegetables and whole grains, while minimizing food high in added sugars and saturated fat suggests that these behaviors are the appropriate focus of intervention.

## 1. Cardiometabolic Risk: A Cluster of Risk Factors

First described by Reaven in 1988 as “Syndrome-X” [1], the constellation of obesity (particularly central or abdominal obesity), high triglycerides, low high density lipoprotein (HDL), elevated blood pressure, and elevated plasma glucose has been variously described as “insulin resistance syndrome”, “metabolic syndrome” and “cardiometabolic risk” (CMR). We use the latter term in this paper to describe the cluster of risk factors. Although the American Diabetes Association, the American Heart Association, and the National Heart, Lung, and Blood Institute at the National Institutes of Health do not recognize the group of risk factors as a syndrome, the organizations all acknowledge that the individual risk factors are causally related to diabetes, heart disease, stroke, and renal failure [2,3]. It is important to understand the origins of CMR and develop appropriate intervention strategies because CMR costs the United States nearly $500 billion a year in health care and lost wages [4,5] and is an emerging problem worldwide.

## 2. CMR: The Association with Obesity

Along the path from health to disease, abdominal obesity and insulin resistance are precursors to elevated blood glucose, dyslipidemia and, possibly, systemic inflammation and hypofibrinolysis [6–14]. When abdominal obesity is present, excess release of circulating free fatty acids and a variety of cytokines that may contribute to inflammation and/or oxidative stress, may cause or exacerbate peripheral insulin resistance [7,8,15–17]. Evidence from cross-sectional and longitudinal studies demonstrates higher risk of morbidity and mortality among individuals with disproportionately high visceral abdominal fat when accounting for total body weight or total body fat [17–19]. Indeed, there is evidence for an association of excess abdominal fat and chronic disease risk across a wide range of BMI values [20–22], and the prevalence of CMR begins to rise well below the cutoff for overweight status (BMI 25 kg/m^2^) [21].

## 3. CMR: The Association with Physical Inactivity

The experimental evidence that exercise modulates insulin resistance and lowers risk factor levels for type 2 diabetes and cardiovascular disease is substantial [23–29]. Well-conducted experiments in men and women of varying race/ethnic groups have clearly demonstrated improvements in insulin sensitivity as a result of single exercise bouts or brief training periods with no change in body weight [24,25,27–37].

Although the precise mechanisms whereby physical activity may have a direct effect on insulin sensitivity are not completely understood, there appears to be support from animal and human studies for mechanisms that involve enhanced insulin receptor and post-receptor function, increased skeletal muscle insulin-sensitive glucose transporter proteins, increased skeletal muscle capillary density, and, over longer time periods, changes in body composition [38–51]. Some of these responses to exercise may also occur in the liver and adipose tissue [36]. Exercise also reduces levels of inflammatory markers, raises high density lipoprotein cholesterol (HDL) and lowers triglycerides.

Through a variety of mechanisms, including those independent of energy balance and weight change, endurance training has been shown to reduce visceral abdominal fat deposition and improve insulin sensitivity [52–55]. Endurance training, such as brisk walking, has been shown to increase HDL [53]. Independent of weight loss, physical activity may reduce blood pressure through a variety of mechanisms, including an attenuation of adrenergic sympathetic activity and a reduction in total peripheral resistance [25,56,57]. Additionally, the reductions in hyperinsulinemia and abdominal fat associated with exercise may reduce blood pressure through decreased sodium retention and reduced sympathetic nervous system stimulation [58–61]. This strong negative association between physical activity and risk of coronary heart disease biologically underscore the plausible effects of physical activity on CMR [30,62].

## 4. CMR: The Association with Dietary Factors

As with exercise, the literature is rich with experimental evidence supporting direct effects of a variety of dietary components on insulin sensitivity and risk factors for cardiovascular disease and diabetes. The results appear to be independent of weight loss. Clinically important effects have appeared within four weeks of intervention in controlled studies [63–65]. It is clear from experimental and observational studies that intake of saturated and *trans*-fatty acids promote insulin resistance, whereas monounsaturated, polyunsaturated, and longer chain n-3 fatty acids appear to improve insulin sensitivity [66–69]. Evidence has emerged over the past decade from experimental studies that less refined and less-starchy carbohydrates, such as fresh fruit, whole grains, green leafy vegetables, nuts, legumes, in contrast to fruit juice, refined grains, and starchy vegetables, have direct and independent beneficial effects on both glucose and insulin metabolism [63,70–95]. Several studies have demonstrated, over the short term, that changing dietary composition without changing calories may improve insulin sensitivity [63,93–97]. There are many plausible physiological explanations for these observations including attenuation of postprandial glycemia, reduced demand for insulin, and direct effects of dietary constituents on beta cells and peripheral insulin receptors [75–81,84,98–103].

The dietary fatty acids that promote insulin sensitivity are the same as those that lower triglycerides and raise HDL. Conversely, low-fat diets that are high in low-quality carbohydrates lower HDL and raise triglycerides. Long-chain fatty acids, such as the n-3 (omega-3) fatty acids found in certain fish, nuts, and seeds, may reduce blood triglyceride concentrations [104–111]. Replacing dietary saturated fat with monounsaturated fat also improves blood lipid profiles [106,112–114]. Controlled feeding studies demonstrate that these dietary effects are independent of changes in body weight [104,112–118].

A diet rich in fruits, vegetables, whole grains, potassium, calcium, and magnesium, and low in sodium may also lower blood pressure [64,119,120]. Although not supported by all studies [121], evidence from observational studies suggests that systemic inflammation and hypofibrinolytic states, conditions known to cluster with the other components of CMR [10–13], can also be ameliorated by following a minimally processed plant-based diet and a physical activity program [81,82,122–124]. Recent cross sectional observations have suggested that certain dietary patterns, such as those including whole grains, fruits, vegetables, low-fat dairy products, and regular physical activity are inversely associated with the odds of having CMR [125–128]. Prospective observations also suggest that these dietary patterns are inversely associated with risk of developing CMR [82,126,129,130].

## 5. CMR: The Evidence from Trials of Diet and Physical Activity

The Oslo Diet and Exercise Study compared the independent and joint effects of diet and exercise (aerobic training three times per week) on changes in CMR among 219 adults [131,132]. The goal of the dietary intervention was weight control, increased fish intake, and decreased fat intake. The exercise intervention included supervised endurance exercise three times per week. The group that received both the diet and physical activity intervention appeared to experience the greatest improvement in risk factors over a one-year period despite weight loss that was modest and no greater than that experienced in the diet only group [131,132]. In fact, the average BMI at follow-up remained in the overweight range (above 25 kg/m^2^). The correlations between changes in body weight and changes in insulin resistance and components of CMR were very weak [132].

Esposito *et al*. conducted a 2-year randomized trial of diet and exercise in 180 adults with CMR [133]. Although changes in activity and body weight (mean difference = −2.8 kg, p < 0.001) were modest over the 2-year intervention, the intervention group experienced significant improvements for all components of CMR. Based on regression analysis, these changes appeared to be largely independent of changes in weight. At the end of the trial, adjusted for weight changes, only 40 of the 90 intervention participants continued to have CMR, compared to 78 of the 90 control participants (p < 0.001).

Four large-scale randomized trials—one of them being the U.S. Diabetes Prevention Program--have examined the effect of diet and exercise in preventing type 2 diabetes among adults with glucose intolerance and high risk for type 2 diabetes [134–137]. These trials were conducted in four different populations—Sweden, China, Finland, and the United States. All found that lifestyle intervention significantly reduced the incidence of type 2 diabetes. These risk reductions were 25% in the Chinese study and 50 to 60% in the other trials. Weight loss was relatively modest across these studies, with means from −0.8% to +1.7% in the control groups and −1.8% to −6.0% in the intervention groups.

If reduction in CMR were dependent on magnitude of weight loss, one would expect correlations between changes in body weight and changes in CMR to be at least moderate in strength. However, all studies showed only weak or no correlations between changes in weight and changes in CMR. The relative risk reduction observed in the U.S. Diabetes Prevention Program for the diet and exercise intervention compared to usual care was ~55% with a mean weight loss of ~6% [135]. The intervention from Sweden and Finland achieved half of the weight loss of the U.S. study, but the relative risks for the diet and exercise intervention compared with usual care in those studies was very similar to the U.S. study. These data from well-conducted randomized trials with precise measures support the hypothesis that the behavioral components themselves, improved diet and increased physical activity, are the primary etiological factors driving the reduction in CMR and risk of diabetes, and that body weight changes may not be required for risk reduction to occur. Even if weight loss is relatively unimportant in the management of CMR, it can be important for other reasons. The same lifestyle behaviors that minimize CMR can aid in preventing weight gain over the long term because physical activity and the diet that prevents CMR also improves energy balance.

The data suggest that the emphasis in such interventions must include lifestyle changes for three reasons: 1) lifestyle changes have known direct effects on risk reduction through a variety of pathways, 2) while good nutrition and adequate physical activity have many benefits in addition to weight control, attempts to lose weight through fasting or unbalanced diets can increase risk of untoward events without the benefits of physical activity or good nutrition, and 3) diet and physical activity are behaviors that are under the direct and immediate control of the individual.

## 6. CMR: Policy Questions

### 6.1. Are Universal Precautions Warranted?

One decision that must be made when designing an intervention program is whether and to what extent to focus on individuals with a particular set of physiological attributes (e.g., weight, BMI, waist-hip ratio, girth, blood pressure, dyslipidemia, and plasma glucose) or to cast a broader net by assessing dietary patterns and physical activity levels for all individuals.

Focusing on physiological parameters is specific and identifies individuals who already have CMR. These individuals are highly likely to go on to develop the complications of CMR − diabetes and cardiovascular disease − and clinical trial evidence demonstrates that, after two years, moderate changes in diet and physical activity levels result in substantial and durable changes in CMR along with an average weight loss of 3% to 6% [138]. However, correction of the physiological abnormalities, and in particular, changing the behaviors that generated CMR in the first place can be expected to be challenging and evidence is equivocal that interventions delivered in a physician’s office result in behavior change [139,140].

On the other hand, a universal lifestyle recommendation for entire communities or populations has been highly successful in some, but not all, instances [141]. Because the majority of Americans already have insulin resistance along with other risk factors [21], most individuals will benefit from responding to a universal recommendation to remain physically active and adopt a diet that is low in saturated fat and high in fruits and vegetables. Interventions delivered over the internet [142] and interventions delivered through the work site [143] have been successful, perhaps because both can increase the reach of the intervention and the frequency with which behavior change support can be delivered.

Fortunately, one strategy doesn’t preclude another, and many organizations endorse the implementation of both strategies simultaneously. The American Heart Association and the National Heart, Lung and Blood Institute jointly state that the “prime emphasis in management of the metabolic syndrome per se is to mitigate the modifiable, underlying risk factors (obesity, physical inactivity, and atherogenic diet) through lifestyle changes [3], and the American Diabetes Association and the European Association for the Study of Diabetes jointly advise that “clinicians should neither rely on, nor require a diagnosis of, metabolic syndrome to prescribe and encourage what is now a fundamental tenet of medicine--weight maintenance (or reduction), exercise, and a healthy meal plan” [144].

A strategy that combines clinical intervention for those at high risk with a universal message of diet and physical activity for everyone could have a large immediate impact on disease rates while potentially shutting down the CMR pipeline. For example, a comprehensive program to prevent and treat heart disease in Finland resulted in a 63% decline in coronary heart disease mortality rates over a 15-year period with concomitant decreases in all-cause mortality and increases in life expectancy [141].

### 6.2. What and How much should be Measured?

A second decision that must be made is the number of screening tests that are performed to characterize CMR in individuals. Because measurement consumes resources that might be used for intervention, program planners must carefully consider this question. Although the pathophysiologic pathways of CMR are complex, neither the assessment tools nor the interventions need to be. Regardless of the intensity of assessment of the individual, prediction of when a particular individual will develop diabetes, heart disease or a stroke remains poor. On the other hand, the presence of the behaviors that convey CMR reduce life expectancy by about 10 years [145], and when smoking and excessive alcohol consumption are added to the risk function, the difference in life expectancy between a low risk and a high risk lifestyle is 14 years [146].

Some physiological components of CMR—weight, height, girth, blood pressure, HDL, triglycerides and glucose—are easily measured at point of care in the clinical setting or by the clinical lab. On the other hand, insulin resistance is not easily measured outside of the research setting. Measuring inflammatory status with high sensitivity C-reactive protein (hs-CRP) conveys little information when other risk factors are taken into account [147,148], and a recent consensus statement from several organizations, including the American Diabetes Association concluded that measuring waist circumference contributes little to the clinical assessment of CMR [149]. Whatever other parameters are assessed, habitual diet and physical activity must be assessed, and they can be assessed with simple checklists [150]. If these tools are used, patients can be provided with immediate feedback and cues to action. A survey of health insurance plans in the United States documents that many health plans are positioned to deliver these behavioral support services [151].

## 7. Conclusions

CMR arises from the constellation of obesity (particularly central or abdominal obesity), high triglycerides, low HDL, elevated blood pressure, and elevated plasma glucose. The mechanism of action appears to be a pathway that starts with a behavior pattern of inadequate physical activity and a diet high in saturated fat and added sugars, and inadequate in fruits, vegetables, and whole grains. These behaviors lead to insulin resistance as an intermediate state. Insulin resistance, appearing even within the “normal” range of BMI (<25 kg/m^2^), leads to the other risk factors in the cluster. These risk factors cause type 2 diabetes mellitus and cardiovascular disease. While maintaining or resuming ideal weight is beneficial for many reasons, experimental evidence demonstrates that improving diet and increasing physical activity reduces CMR independent of the degree of weight loss. These observations, and the fact that individuals can act directly to change their diet and increase their physical activity, suggest that the most effective intervention strategy to prevent and treat CMR is a universal intervention focusing on a healthy diet, adequate physical activity, and prevention of weight gain in adults and excess weight gain in youth.
